# Cerebral amyloid angiopathy: A clinicopathological study of three cases

**DOI:** 10.4103/0972-2327.70879

**Published:** 2010

**Authors:** Jalesh N. Panicker, D. Nagaraja, Yasha T. Chickabasaviah

**Affiliations:** Department of Neurology, National Institute of Mental Health and Neurosciences, Bangalore - 560 029, India; 1 Department of Neuropathology, National Institute of Mental Health and Neurosciences, Bangalore - 560 029, India

**Keywords:** Beta amyloid, cerebral amyloid angiopathy, intracerebral hemorrhage, senile plaques

## Abstract

Cerebral amyloid angiopathy (CAA) is an important cause for intracerebral hemorrhage (ICH), yet often goes undiagnosed in the absence of histological examination of the blood vessels in the clot. In this study, we report three patients who presented with ICH. Two patients had no risk factors for bleed, whereas one patient had systemic hypertension. Tissue for analysis was obtained during hematoma evacuation in two patients and necropsy in the third. Histopathology in all three patients revealed severe degree of amyloid angiopathy with extensive amyloid deposits in the vessel walls, which was diagnostic of CAA. Both medium- and small-sized leptomeningeal and cortical vessels were affected. The vascular amyloid deposits stained with Congo red and displayed characteristic birefringence under polarizing light. In addition, vessels also showed fibrinoid necrosis and vascular endothelial proliferation. Immunohistochemistry demonstrated beta-amyloid peptide in all three cases—the protein most commonly involved in sporadic CAA. Senile plaques with amyloid cores were present in all areas, whereas neurofibrillary tangles were restricted to the medial temporal region in the autopsied case. CAA is an important cause for intracerebral bleed and may be a contributory factor even when other risk factors for ICH are present. Areas of hemorrhage tend to correlate with severity of CAA changes.

## Introduction

Intracerebral hemorrhage (ICH) accounts for 10–20% of all strokes.[1] Cerebral amyloid angiopathy (CAA) as a cause for bleed often goes undiagnosed and is frequently overlooked.[2] Patients characteristically present with recurrent lobar ICH, and the hallmark of this condition is amyloid deposition affecting the leptomeningeal and cortical arteries, arterioles, and capillaries.[3] We report three cases of ICH due to CAA, and review its neuropathological features.

## Case Reports

Our hospital is a tertiary referral center for the management of patients with acute neurological illness catering to a population spread over four states in south central India. Critical patients with ICH are admitted to a dedicated stroke unit where neurological parameters are closely monitored for level of consciousness using the Glasgow Coma Scale (GCS), pupil size, and development of new neurological deficits. Initial imaging is with cranial CT scan and patients showing deterioration on medical management are serially imaged and undergo surgical decompression by craniotomy under general anesthesia. Evacuated hematomas are sent for detailed pathological evaluation.

### Case 1

**Figure 1:**
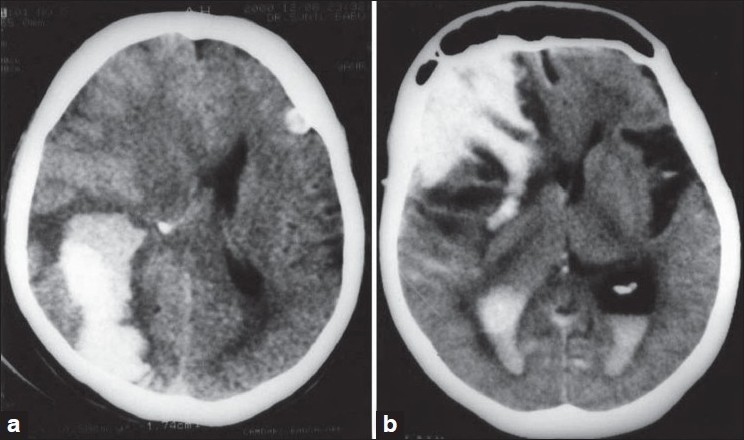
CT scans demonstrating (a) Case 1: Acute lobar hematoma in the right occipital lobe. There is evidence of mild perilesional edema and mass effect with effacement of ipsilateral lateral ventricle and midline shift. Another small cortical bleed is seen in the left frontal lobe. (b) Case 2: Acute right frontal hematoma with intraventricular extension. Perilesional edema is noted with midline shift

A 78-year-old man was admitted with a history of sudden-onset of loss of consciousness. However, his medical history did not reveal any risk factors for ICH such as systemic hypertension, trauma, or use of medications, and there was no history of cognitive impairment. Blood pressure was 160/100 mmHg. He had a score of eight (E2M4V2) on the GCS and was noted to have right-sided hemiplegia, bilateral extensor plantar response, and asymmetric pupillary reflex. Cranial CT scan showed an 11 cm × 5 cm lobar hematoma in the right occipital lobe causing midline shift, and a smaller cortical bleed in the left posterior frontal lobe [Figure 1a]. Routine blood tests were normal, including coagulation profile. Surgical decompression was deferred due to poor general condition, and he expired. A partial autopsy was carried out, restricted to removal of the brain.

### Case 2

A 67-year-old woman was admitted with a history of sudden-onset of altered sensorium and paucity of movements of the left side of the body, following a day of diarrhea and vomiting. She had been on regular treatment for systemic hypertension and diabetes mellitus for 10 years. There was no history to suggest a bleeding disorder. Moreover, there had not been a history of cognitive decline. Blood pressure was 170/100 mmHg. She had a score of 10 (E2M5V3) on the GCS and was noted to have left-sided hemiparesis, bilateral extensor plantar response, and symmetric pupillary reflex. Cranial CT scan showed a 10 cm × 7 cm hematoma in the right frontal lobe with midline shift and extension into the lateral ventricles and subarachnoid space [Figure 1b] without contrast enhancement. ICH secondary to systemic hypertension, or possibly cerebral venous thrombosis in view of preceding diarrhoea, was considered. Investigations showed haemoglobin 7.3 g/dL, normocytic normochromic anemia, and ESR 40 mm in the first hour. Blood sugar was 400 mg/dL, blood urea 68 mg/dL, and serum creatinine 1.4 mg/dL. Other laboratory parameters were normal, including coagulation profile. Deteriorating level of consciousness prompted emergent right frontotemporal craniotomy and evacuation of hematoma. Repeat CT scan showed reduced midline shift and bilateral subdural collections. In spite of an initial improvement in neurological status, she developed progressively worsening renal failure and expired. Autopsy was not performed.

### Case 3

A 65-year-old man presented with sudden-onset of headache followed by loss of consciousness. His medical history did not reveal any risk factors for ICH, and there was no history of cognitive impairment. Blood pressure was 180/110 mmHg. He had a score of eight (E2M4V2) on the GCS and was noted to have left-sided hemiplegia and asymmetric pupillary reflex. Cranial CT scan showed a 4 cm × 5 cm hematoma in the right parietal lobe with surrounding edema and minimal midline shift. Routine blood tests were normal, including coagulation profile. The cause for ICH could not be established, though the possibility of previously undetected systemic hypertension was considered. Level of consciousness gradually deteriorated, and he underwent emergent right parietal craniotomy and hematoma evacuation. Postoperatively, he moved all four limbs and followed simple commands. However, on the tenth postoperative day, he developed alteration of sensorium once again and repeat cranial CT showed a recollection of blood in the right parieto-occipital region. The hematoma was evacuated once again, his condition improved and he was discharged.

The clinical features of the three cases are summarized in Table 1.

**Table 1:** Summary of clinical features

Case	Age/Gender	Presenting feature	Site of hemorrhage	Risk factors for ICH	Clinical impression	Hospital course
1	78/M	Unconsciousness, rieht hemiplegia	Right occipital, left frontal	No	ICH? cause	Conservative management; expired
2	67/F	Altered sensorium, left hemiparesis	Right frontal	Yes (systemic hypertension)	ICH ? Cerebral venous thrombosis	Hematoma evacuation; expired from medical conditions
3	65/M	Headache, unconsciousness, left hemiplegia	Right parietal	No	ICH ? cause	Hematoma evacuation; recollection on tenth postoperative day evacuated; discharged

ICH, Intracerebral hemorrhage.

### Pathological study

Formalin-fixed autopsied brain was analyzed in Case 1, and the evacuated hematomas were examined in Cases 2 and 3. Formalin-fixed paraffin sections were stained with hematoxylin–eosin, Congo red, and modified Bielschowsky silver stain. Sections stained with Congo red were examined under crossed polarized light for analyzing vascular amyloid, amyloid cores of mature neuritic senile plaques (SP) and neurofibrillary tangles (NFT). Representative sections from the three cases were immunostained by indirect immunoperoxidase technique using antibodies to amyloid protein (polyclonal, 1:100, antiserum 1280, gift of Dr. Dennis Selkoe, USA), hyperphosphorylated tau (monoclonal, 1:50, PHF-1, gift of Dr. Sharon Greenberg, USA), and ubiquitin (monoclonal, 1:100, clone 3–39, donated by Dr. Grundke-Iqbal, USA). The immunoreaction was visualized by horseradish peroxidase tagged secondary antibody with DAB/H_2_O_2_, incorporating appropriate positive and negative controls. Vascular amyloid changes were visually graded as mild, moderate, or severe [Table 2].[4] A detailed study of the topographic distribution of the vascular changes and additional Alzheimer-type pathology was possible in Case 1.

**Table 2:** Grading of vascular amyloid changes proposed by Vonsattel *et al*.[4]

Mild CAA	Thin rim of congophilia around normal or atrophic smooth muscle fi bers in the media of an otherwise normal vessel
Moderate CAA	Media replaced by amyloid and thickened, no evidence of remote or recent blood leakage
Severe CAA	Extensive amyloid deposition, focal fragmentation of vessel wall, and at least one focus of perivascular leakage and fibrinoid necrosis

CAA, Cerebral amyloid angiopathy

## Results

### Gross pathology

#### Case 1

The autopsy brain showed diffuse, subarachnoid hemorrhage over both cerebral hemispheres, maximally over the left frontal region, extending to the base along the Sylvian fissures. Coronal slices revealed two lobar hemorrhages centered in the white matter of the left frontal and right parieto-occipital regions. Both extended into the subarachnoid space and the former into the lateral ventricle as well. There was marked downward herniation and brainstem compression. The cerebral vessels forming the circle of Willis showed moderate degrees of atherosclerosis.

#### Cases 2 and 3

Evacuated hematomas showed blood clots and few hemorrhagic cortical fragments partially covered by leptomeninges.

### Histopathology

#### Case 1

**Figure 2:**
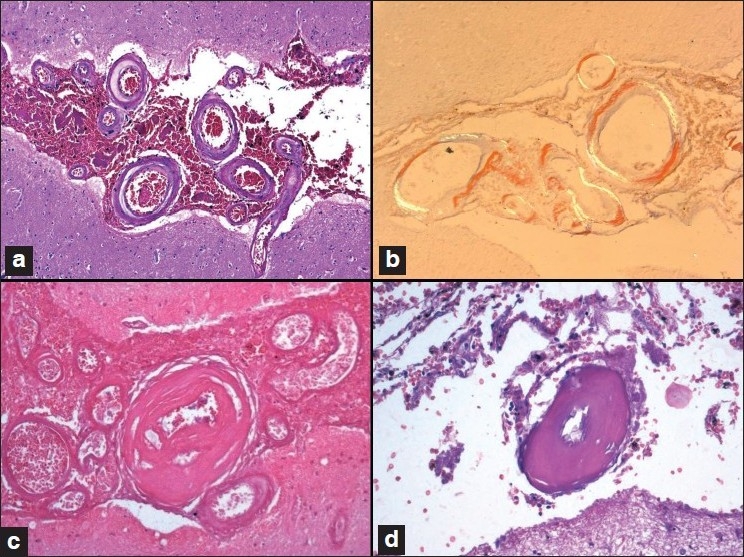
(a and b) A cluster of rigid contoured and thickened leptomeningeal vessels embedded in a pool of subarachnoid hemorrhage (a) exhibit apple-green birefringence on Congo-red stain and polarized light (b).(c and d) Severe amyloid angiopathy with extensive vascular amyloid deposits, fi broid necrosis, luminal narrowing, and fragmentation of the vessel wall (a, c, and d: H and E stain; b: Congo-red stain; original magnifi cation a, b, and c: ×40; d: ×80).

**Figure 3:**
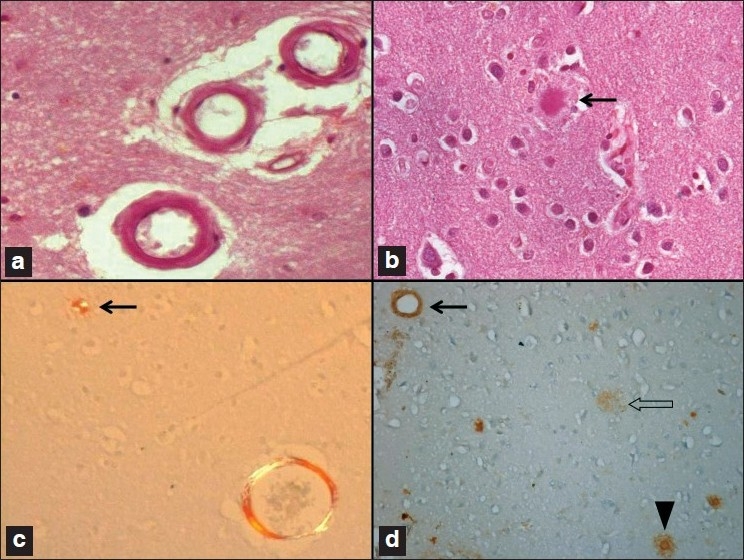
(a and c) Superfi cial cortical, penetrating arterioles with rigid round profi les and amorphous eosinophilic amyloid deposits in the vessel walls which are congophilic and birefringent (c). (b) An amyloid senile plaque (arrow) as seen in a hematoxylin and eosin stained section. It demonstrates Maltese cross birefringence with Congo red (c, arrow). (d) Beta-amyloid staining labels the vessels (arrow), the diffuse amyloid plaque (open arrow), and the cored mature plaque (arrowhead) (a and b: H and E stain; c: Congo-red stain; d: betaamyloid immunostaining; original magnifi cation, a and c: ×160, b: ×320, d: ×80).

Sections showed extensive intraparenchymal and subarachnoid hemorrhage in the right occipital and left frontal regions. The striking histological feature was the rigid contour of the leptomeningeal arteries and arterioles in the subarachnoid space amidst the hemorrhage [Figure 2a and c]. The penetrating superficial cortical parenchymal arterioles similarly showed round and thickened vascular contours [Figure 3a]. The vascular thickening resulted from amyloid deposits that were homogenous, amorphous, eosinophilic, and stained positive with Congo red and revealed apple-green birefringence under crossed polarized light [Figures 2b and 3c]. Several vessels showed fibrinoid necrosis of the vessel wall, endothelial cell prominence, and occasional fragmentation of the media and distortion due to rupture [Figure 2]. Features suggested severe vascular involvement [Table 2]. Strong immunostaining of the vessel wall established the chemical nature of the amyloid to be beta-amyloid [Figure 3d].

The vascular changes involved both the leptomeningeal and superficial cortical vessels. Vessels in the deep cortical layers and the white matter were unaffected. Severe grade of vascular pathology was seen in all the lobes of the cerebral hemispheres with no preferential involvement of any one lobe. There was no significant difference in the severity of changes in the areas with hematoma and those without, although occasional ruptured vessels could be seen in the former. In contrast, vascular changes within the cerebellum and overlying leptomeninges were of a moderate degree. In the brainstem, the amyloid angiopathy was of mild degree. Basal ganglia and thalamus were devoid of vascular amyloid deposits.

In addition to the vascular pathology, many diffuse beta-amyloid plaques [Figure 3d] and mature plaques with Congo-red positive dense amyloid cores [Figure 3c] were seen in the neuropil and though noted in all areas of the cerebral cortex, were maximally seen in the hippocampus and medial temporal lobe. The proportion of mature plaques was also greater in this region. No plaques were found in the cerebellum and brain stem. NFT were found to be restricted to the medial temporal region involving the subiculum and entorhinal cortex.

**Figure 4:**
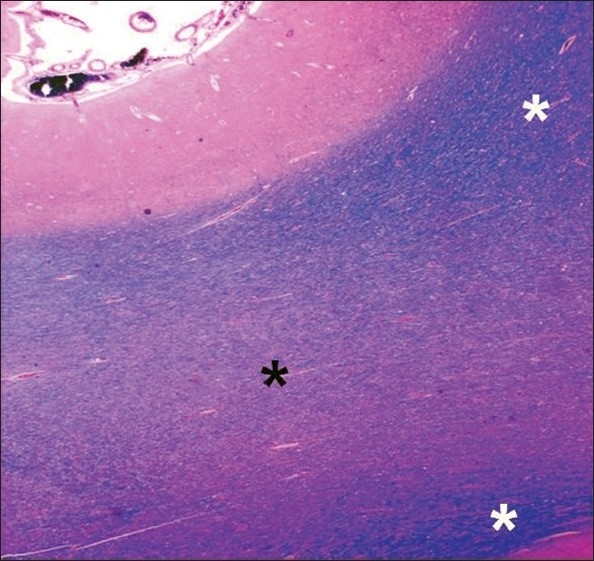
Scanner view of gray and white matter showing white matter pallor (black asterisk). Compare with normal myelin (white asterisk) (Luxol fast blue stain for myelin; original magnifi cation, ×16).

There was mild-to-moderate periventricular spongiosus and white matter pallor [Figure 4]. Two small, old, linear infarcts about half a centimeter in size were detected in the periventricular area of the left temporal lobe.

#### Cases 2 and 3

The meningo-cortical vessels showed features to suggest severe amyloid angiopathy [Table 2] with extensive areas of subarachnoid and intraparenchymal hemorrhage. Some of the leptomeningeal vessels were significantly thickened with occasional ruptures. Fibrin deposition and luminal narrowing were noted. A few amyloid plaques were present in the neuropil. There were no NFT.

## Discussion

Although CAA is an important cause for ICH, the diagnosis is often missed, as confirmation requires special pathological studies. In the present study, the cause for ICH could not be established preoperatively and CAA had not been considered at the stage of the clinical evaluation. The diagnosis was established only on examination of the evacuated hematomas or autopsy specimen. Although systemic hypertension was an evident risk factor in Case 2, CAA was of severe grade and is likely to have contributed to the development of ICH.[5] The hemorrhagic manifestations of CAA characteristically include recurrent or multiple lobar hemorrhages that are cortical and subcortical in location. Recurrence of bleed was seen in Case 3, and multiple hemorrhages were seen in Case 1. Subarachnoid hemorrhage is a frequent association and may result from direct extension of intraparenchymal bleed or rupture of affected leptomeningeal vessels.[6,7] The initial manifestation may be transient neurological deficits, characterized by numbness, paraesthesias, or weakness, which result from cortical microbleeds, focal subarachnoid hemorrhages, or lacunar infarcts[6] and can be identified using gradient-echo MRI sequences.[8] Angiography is usually normal, and there are no serum or CSF markers for CAA.[9]

The distribution of amyloid change in Case 1 was typical. Though changes were seen diffusely, ruptured vessels were restricted to the sites of hemorrhage. Changes are most commonly seen in the occipital[10] and frontal lobes.[11] Cerebellum, brainstem, and subcortical white matter are infrequently involved and are areas with low incidence for hemorrhage.[12] Thalamus and basal ganglia are usually devoid of changes. This is in contrast to the pattern of vascular involvement associated with hypertensive arteriosclerotic and lipohyaline changes.[13]

Beta-amyloid peptide, a cleavage product of beta-amyloid precursor protein, is the amyloidogenic protein deposited in almost all cases of sporadic CAA. Impaired clearance of beta-amyloid from the brain interstitial fluid space is postulated to be the cause of amyloid deposition in the vessel wall and parenchyma. With advancing age, there is increased vascular fibrosis, loss of smooth muscle cells, and reduced pulsations that hamper the elimination of soluble beta-amyloid.[14] Deposits are initially distributed at the sites of vessel bifurcation and with progressive disease, become more diffuse, resulting in microaneurysms and fibrinoid necrosis.[4,15] All three cases had severe vascular amyloid changes, characterized by extensive amyloid deposits, vessel wall fragmentation, fibrinoid necrosis, and endothelial cell proliferation.[4] Occurrence of ICH has been consistently associated with severe angiopathy.[4] Preferential involvement of superficial vessels results in lobar hematomas and subarachnoid hemorrhage, whereas widespread involvement of vessels accounts for recurrent and even simultaneous bleeds.[4,16] Most cases of CAA may show neuritic SP widely distributed in the neuropil.[4] as had been demonstrated in Case 1. However, NFT were restricted to the medial temporal area, a finding consistent with normal aging.[17] In addition to vascular changes, histopathology may also reveal inflammation and this may suggest a potential role for immunomodulatory therapy in a subset of patients with CAA.[18]

CAA may occur in elderly individuals and patients with Alzheimer’s disease (AD) without developing ICH. It has been reported to occur in 10–40% of all elderly brains and 80% of brains with concomitant AD.[19] At this center, an autopsy study of 52 cases revealed the incidence of CAA in the population above 60 years to be 30% (unpublished observations). These findings suggest that there may be other factors contributing to the development of hemorrhage.[4,20,21] Recently, CAA has also been linked to the vascular cognitive impairment due to involvement of the microvasculature and extensive white matter changes.[15,22] The patient presented in Case 1 showed white matter changes and a lacunar infarct. Although there was no history of cognitive decline, more subtle cognitive deficits could be missed unless formally assessed by neuropsychiatric tests.

In conclusion, CAA is an important cause for intracerebral bleed and may be a contributory factor when other risk factors for ICH are present. This series highlights the need to consider the diagnosis of CAA in patients presenting with cortical-subcortical lobar hematomas, especially when multiple or recurrent. Areas of hemorrhage tend to correlate with severity of CAA changes.

## References

[1] Feigin VL, Lawes CM, Bennett DA, Anderson CS (2003). Stroke epidemiology: a review of population-based studies of incidence, prevalence, and case-fatality in the late 20th century. Lancet Neurol.

[2] Ariesen MJ, Claus SP, Rinkel GJ, Algra A (2003). Risk Factors for Intracerebral Hemorrhage in the General Population: A Systematic Review. Stroke.

[3] McCarron MO, Nicoll JA, Ironside JW, Love S, Alberts MJ, Bone I (1999). Cerebral amyloid angiopathy-related hemorrhage. Interaction of APOE ε2 with putative clinical risk factors. Stroke.

[4] Vonsattel JP, Myers RH, Hedley-Whyte ET, Ropper AH, Bird ED, Richardson EP (1991). Cerebral amyloid angiopathy without and with cerebral hemorrhages: A comparative histological study. Ann Neurol.

[5] Ritter MA, Droste DW, Hegedus K, Szepesi R, Nabavi DG, Csiba L (2005). Role of cerebral amyloid angiopathy in intracerebral hemorrhage in hypertensive patients. Neurology.

[6] Maia LF, Mackenzie IR, Feldman HH (2007). Clinical phenotypes of Cerebral Amyloid Angiopathy. J Neurol Sci.

[7] Takeda S, Yamazaki K, Miyakawa T, Onda K, Hinokuma K, Ikuta F (2003). Subcortical hematoma caused by cerebral amyloid angiopathy: does the first evidence of hemorrhage occur in the subarachnoid space?. Neuropathology.

[8] Viswanathan A, Chabriat H (2006). Cerebral microhemorrhage. Stroke.

[9] Greenberg SM, Edgar MA (1996). Cerebral hemorrhage in a 69-year old woman receiving warfarin. N Engl J Med.

[10] Attems J (2005). Sporadic cerebral amyloid angiopathy: pathology, clinical implications, and possible pathomechanisms. Acta Neuropathol.

[11] Xu D, Yang C, Wang L (2003). Cerebral amyloid angiopathy in aged Chinese: a clinico-neuropathological study. Acta Neuropathol.

[12] Masuda J, Tanaka K, Ueda K, Omae T (1988). Autopsy study of incidence and distribution of cerebral amyloid angiopathy in Hisayama, Japan. Stroke.

[13] Thal DR, Ghebrenedhin E, Orantes M, Wiestler OD (2003). Vascular pathology in Alzheimer disease: correlation of cerebral amyloid angiopathy and arteriosclerosis/lipohyalinosis with cognitive decline. J Neuropathol Exp Neurol.

[14] Weller RO, Subash M, Preston SD, Mazanti I, Carare RO (2008). Perivascular drainage of amyloid-beta peptides from the brain and its failure in cerebral amyloid angiopathy and Alzheimer’s disease. Brain Pathol.

[15] Greenberg SM, Gurol ME, Rosand J, Smith EE (2004). Amyloid angiopathy-related vascular cognitive impairment. Stroke.

[16] Johnson KA, Gregas M, Becker JA, Kinnecom C, Salat DH, Moran EK (2007). Imaging of Amyloid Burden and Distribution in Cerebral Amyloid Angiopathy. Ann Neurol.

[17] Yasha TC, Shankar L, Santosh V, Das S, Shankar SK (1997). Histopatho-logical and immunohistochemical evaluation of ageing changes in normal human brain. Indian J Med Res.

[18] Kinnecom C, Lev MH, Wendell L, Smith EE, Rosand J, Frosch MP (2007). Course of cerebral amyloid angiopathy–related inflammation. Neurology.

[19] Jellinger KA (2002). Alzheimer disease and cerebrovascular pathology: An update. J Neural Transm.

[20] Greenberg SM, Rebeck GW, Vonsattel JPG, Gomez-Isla T, Hyman BT (1995). Apolipoprotein E ε4 and cerebral hemorrhage associated with amyloid angiopathy. Ann Neurol.

[21] Sanchez-Valle R, Llado A, Ezquerra M, Rey MJ, Rami L, Molinuevo JL (2007). A novel mutation in the PSEN1 gene (L286P) associated with familial early-onset dementia of Alzheimer type and lobar haematomas. Eur J Neurol.

[22] Knopman DS (2007). Cerebrovascular disease and dementia. Br J Radiol.

